# Supplementary material for: A Likelihood Approach for Real-Time Calibration of Stochastic Compartmental Epidemic Models
Source: PLoS Comput Biol. 2017 Jan 17;13(1):e1005257. doi: 10.1371/journal.pcbi.1005257 (PMC5240920; doi:10.1371/journal.pcbi.1005257)
Supplement: S1 File — (TAR.GZ) [file pcbi.1005257.s014.tar.gz › HSPH_Online-SI-Revision/PF08/n1-mild/n1-mild_PF_table-8.pdf]

1	R0	{1.27677, 7.89375}	{1.78514, 13.0383}	{{1.08887, 6.8071}, {2.51743, 18.6082}}	{46.1645, 72.6265}	inf	9.	75.5457	{4.99197, 164.545}	755.768	0.798372	newly	11.	42.2	{3., 91.}	309.455	0.591005	Reff	1.2423	1.32731	{0.843719, 1.91401}	26.5215	ar	400.	620.07	{172., 901.}	76.1575
2	R0	{1.33231, 10.5259}	{1.64404, 13.0249}	{{1.13176, 6.31117}, {2.22212, 19.0269}}	{30.059, 42.1567}	inf	43.	67.7592	{3.53587, 143.199}	109.293	0.460967	newly	32.	33.05	{4., 63.}	64.5313	0.405759	Reff	1.07517	0.976912	{0.640408, 1.33161}	24.6884	ar	467.	586.8	{238., 834.}	48.2056
3	R0	{1.2273, 6.76944}	{1.96744, 11.5664}	{{1.29991, 5.3704}, {2.63959, 17.3398}}	{61.1559, 79.2633}	inf	19.	39.0575	{3.8895, 91.6036}	146.597	0.458282	newly	19.	42.77	{2., 100.}	177.737	0.506777	Reff	1.19048	1.79811	{1.19829, 2.48668}	52.7754	ar	314.	676.57	{278., 904.}	128.965
4	R0	{1.41215, 17.5742}	{1.74671, 13.133}	{{1.11461, 5.62019}, {2.75598, 19.0269}}	{34.7087, 28.9363}	inf	19.	67.6378	{3.85453, 155.247}	290.685	0.61115	newly	6.	35.03	{3., 74.}	503.167	0.751131	Reff	1.34578	1.27688	{0.77803, 1.96011}	30.0886	ar	465.	592.25	{220., 913.}	54.8882
5	R0	{1.42222, 6.15886}	{1.8472, 13.3347}	{{1.11164, 6.62607}, {2.7943, 19.0443}}	{40.5622, 119.554}	inf	13.	79.6893	{6.295, 188.089}	528.778	0.698981	newly	24.	45.24	{4., 98.}	130.833	0.45356	Reff	1.34115	1.38471	{0.872175, 2.10045}	27.6425	ar	490.	621.77	{175., 919.}	53.4673
6	R0	{1.24363, 9.95596}	{1.94297, 11.4712}	{{1.27357, 5.3704}, {2.63959, 17.3398}}	{57.2002, 39.8397}	inf	3.	38.4204	{2.59214, 90.7609}	1192.67	0.942114	newly	3.	44.79	{2., 102.}	1409.67	0.937486	Reff	1.23741	1.77518	{1.18566, 2.48668}	46.0072	ar	411.	658.55	{233., 902.}	79.5109
7	R0	{1.36478, 5.96056}	{1.99096, 11.9743}	{{1.21457, 5.04943}, {2.79269, 18.9406}}	{50.5512, 107.914}	inf	14.	54.7564	{4.18043, 120.356}	315.847	0.569767	newly	23.	49.63	{3., 114.}	166.043	0.514472	Reff	1.32384	1.71239	{1.01577, 2.43299}	39.6794	ar	484.	681.66	{268., 924.}	61.405
8	R0	{1.36335, 17.6429}	{1.73059, 14.6851}	{{1.25149, 7.75913}, {2.37305, 20.}}	{30.4152, 23.6237}	inf	13.	87.2225	{27.191, 164.18}	570.942	0.732178	newly	3.	42.85	{16., 74.}	1328.33	1.089	Reff	1.33472	1.20066	{0.771626, 1.72068}	22.9083	ar	484.	777.89	{535., 1014.}	62.1012
9	R0	{1.26575, 5.28707}	{1.68, 14.7098}	{{1.2158, 8.24495}, {2.30429, 20.}}	{34.3986, 180.464}	inf	7.	84.4703	{28.7749, 160.125}	1106.72	0.992141	newly	8.	39.7	{15., 67.}	396.5	0.633731	Reff	1.13918	1.11106	{0.781341, 1.44471}	21.1718	ar	472.	757.19	{494., 979.}	62.5996
10	R0	{1.42942, 9.49905}	{1.71403, 13.9195}	{{1.23758, 7.44164}, {2.32891, 20.}}	{26.4726, 56.8702}	inf	25.	83.8432	{24.6436, 169.211}	240.104	0.43148	newly	22.	42.42	{14., 75.}	108.545	0.292283	Reff	1.26932	1.14998	{0.721718, 1.46533}	21.3851	ar	468.	766.57	{498., 1008.}	65.1987
11	R0	{1.46827, 15.0983}	{1.65586, 13.4322}	{{1.20353, 4.61694}, {2.37785, 20.}}	{23.555, 30.6461}	inf	7.	80.186	{13.0847, 182.475}	1047.17	0.901846	newly	4.	37.71	{13., 76.}	842.75	0.892515	Reff	1.43009	1.07507	{0.706812, 1.39041}	28.3665	ar	495.	726.51	{475., 990.}	49.6949
12	R0	{1.25751, 13.279}	{1.65991, 14.5137}	{{1.24086, 7.41316}, {2.3417, 20.}}	{32.9677, 31.1133}	inf	9.	76.9995	{25.2374, 182.744}	756.062	0.821257	newly	6.	37.47	{15., 68.}	524.5	0.720504	Reff	1.20847	1.15485	{0.765899, 1.44219}	19.7682	ar	366.	737.75	{509., 972.}	101.571
13	R0	{1.3425, 9.16361}	{1.66352, 12.9503}	{{1.11228, 5.71232}, {2.24606, 19.0443}}	{31.4023, 58.32}	inf	36.	72.3707	{2.39468, 158.178}	148.75	0.534198	newly	33.	35.85	{3., 72.}	67.	0.438066	Reff	1.12233	1.013	{0.648025, 1.4114}	24.5276	ar	442.	580.66	{202., 855.}	54.0136
14	R0	{1.18936, 6.20583}	{1.75537, 12.9009}	{{1.07662, 6.62607}, {2.73737, 18.2815}}	{51.3414, 111.668}	inf	20.	72.2901	{6.295, 168.25}	286.584	0.540888	newly	20.	41.89	{5., 94.}	149.15	0.440931	Reff	1.1287	1.34379	{0.894697, 2.0885}	28.834	ar	382.	594.02	{172., 910.}	77.0052
15	R0	{1.27137, 9.78868}	{1.82933, 13.2444}	{{1.09005, 6.62607}, {2.79269, 19.0443}}	{49.3962, 47.4112}	inf	14.	78.1273	{5.87089, 188.089}	476.283	0.672576	newly	12.	44.45	{4., 100.}	293.917	0.564624	Reff	1.20017	1.37726	{0.894697, 2.10045}	29.2642	ar	496.	612.78	{172., 914.}	52.625
16	R0	{1.23102, 13.6452}	{1.82466, 13.6388}	{{1.14345, 6.31117}, {2.59907, 19.1994}}	{51.1142, 26.9147}	inf	23.	73.05	{3.85453, 165.044}	250.606	0.567401	newly	11.	37.4	{4., 75.}	263.273	0.600506	Reff	1.16085	1.31333	{0.77803, 1.96045}	35.0747	ar	445.	642.98	{246., 907.}	63.5461
17	R0	{1.27213, 13.7452}	{1.83545, 13.3739}	{{1.10693, 6.74711}, {2.64373, 19.0269}}	{49.1429, 24.1261}	inf	8.	60.5667	{3.69964, 155.006}	674.175	0.757987	newly	5.	35.23	{2., 82.}	622.2	0.768606	Reff	1.2505	1.51132	{0.961356, 2.18291}	33.5262	ar	367.	643.61	{236., 908.}	93.6866
18	R0	{1.25318, 11.2151}	{1.65685, 14.6872}	{{1.19194, 7.42139}, {2.20487, 20.}}	{33.9278, 44.6543}	inf	12.	87.0236	{25.1331, 184.984}	627.349	0.745852	newly	5.	39.8	{16., 83.}	696.	0.817506	Reff	1.17924	1.09933	{0.752541, 1.34558}	20.7236	ar	416.	744.2	{518., 989.}	79.375
19	R0	{1.46006, 8.11496}	{1.83931, 13.3579}	{{1.10693, 6.74711}, {2.64373, 19.0269}}	{37.1288, 71.2872}	inf	12.	60.1442	{3.69964, 155.006}	424.075	0.637318	newly	15.	35.01	{2., 82.}	169.4	0.489972	Reff	1.43232	1.51632	{0.961356, 2.18291}	27.7989	ar	496.	644.13	{236., 908.}	55.3286
20	R0	{1.14273, 6.71261}	{1.73717, 13.208}	{{1.11228, 5.62019}, {2.75598, 19.0443}}	{53.84, 104.542}	inf	10.	68.0562	{3.85453, 162.199}	604.613	0.767404	newly	11.	35.49	{3., 80.}	250.818	0.586459	Reff	1.10273	1.26183	{0.77803, 1.96011}	32.3179	ar	362.	590.54	{220., 913.}	83.4972
21	R0	{1.42432, 8.23905}	{1.77268, 12.9085}	{{1.03515, 5.72868}, {2.67299, 18.8445}}	{38.39, 70.9723}	inf	12.	73.9298	{3.85453, 182.522}	542.258	0.742925	newly	16.	37.53	{3., 82.}	171.188	0.528487	Reff	1.21067	1.20179	{0.731217, 1.9251}	32.4879	ar	405.	598.13	{179., 906.}	74.2247
22	R0	{1.29386, 17.863}	{1.79936, 13.2178}	{{1.11228, 6.19362}, {2.73737, 18.4249}}	{44.3435, 28.0609}	inf	15.	75.2396	{6.98202, 159.843}	418.589	0.614909	newly	3.	43.85	{5., 94.}	1368.33	0.99959	Reff	1.26151	1.34897	{0.862846, 1.99815}	26.0156	ar	488.	619.23	{197., 903.}	51.8217
23	R0	{1.39274, 13.0167}	{1.72405, 14.307}	{{1.23907, 7.36754}, {2.46062, 20.}}	{29.8728, 32.9597}	inf	20.	83.7044	{23.489, 176.547}	319.433	0.512275	newly	9.	40.25	{15., 75.}	347.222	0.583821	Reff	1.22283	1.1257	{0.774372, 1.4673}	22.4833	ar	475.	762.87	{501., 1017.}	63.3958
24	R0	{1.32178, 15.8991}	{1.60886, 13.0255}	{{1.11365, 5.98888}, {2.07206, 18.9813}}	{28.9335, 27.3593}	inf	13.	61.1035	{2.57939, 140.409}	396.307	0.642097	newly	5.	33.21	{3., 69.}	583.	0.758186	Reff	1.27023	1.14284	{0.769594, 1.59714}	25.2054	ar	471.	567.9	{229., 815.}	46.811
25	R0	{1.37568, 17.8427}	{1.70562, 14.7325}	{{1.2057, 8.96949}, {2.37313, 20.}}	{29.1616, 23.3058}	inf	15.	87.3823	{26.4736, 170.926}	484.802	0.664992	newly	9.	40.58	{15., 78.}	350.889	0.571331	Reff	1.2615	1.08304	{0.6779, 1.49489}	24.4683	ar	398.	757.21	{498., 1018.}	90.2538
26	R0	{1.20993, 6.4656}	{1.67286, 14.038}	{{1.11228, 6.0993}, {2.30371, 19.2972}}	{41.9897, 122.917}	inf	16.	70.9683	{2.48733, 160.24}	376.663	0.666549	newly	14.	35.08	{1., 76.}	189.857	0.548901	Reff	1.13007	1.13637	{0.762641, 1.56866}	23.6787	ar	345.	589.42	{245., 870.}	84.858
27	R0	{1.20548, 13.8003}	{1.66632, 13.7642}	{{1.20371, 6.40764}, {2.49125, 20.}}	{38.8975, 30.5713}	inf	23.	79.3554	{13.0924, 182.733}	258.834	0.485352	newly	9.	36.68	{12., 74.}	310.222	0.537301	Reff	1.15003	1.03797	{0.646194, 1.32666}	21.749	ar	350.	728.68	{461., 1004.}	108.606
28	R0	{1.3176, 14.6594}	{1.78536, 13.277}	{{1.07662, 5.83416}, {2.73737, 18.8228}}	{41.9832, 25.6123}	inf	4.	70.5031	{10.6529, 158.864}	1666.67	1.06291	newly	1.	43.2	{5., 90.}	4222.	1.43015	Reff	1.30442	1.35844	{0.862846, 1.99815}	26.3616	ar	362.	619.91	{197., 902.}	88.9751
29	R0	{1.28202, 6.23047}	{1.62349, 13.0681}	{{1.11365, 5.98888}, {2.24606, 18.9813}}	{32.4399, 114.97}	inf	7.	62.4215	{2.45373, 146.457}	812.446	0.834399	newly	13.	33.66	{1., 69.}	195.385	0.533622	Reff	1.22561	1.11102	{0.760526, 1.56866}	25.6124	ar	391.	575.5	{245., 861.}	64.3018
30	R0	{1.28177, 8.83252}	{1.80091, 13.2715}	{{1.10693, 6.62607}, {2.60082, 19.0269}}	{45.8494, 59.7278}	inf	15.	58.637	{3.4726, 150.177}	319.798	0.592875	newly	15.	34.08	{2., 80.}	163.867	0.5042	Reff	1.24332	1.48578	{0.961356, 2.1477}	32.4536	ar	427.	630.71	{207., 898.}	71.
31	R0	{1.35886, 5.97708}	{1.83655, 13.3685}	{{1.05743, 7.10524}, {2.73517, 17.5057}}	{43.6595, 126.207}	inf	20.	90.5356	{7.16405, 212.929}	377.899	0.625802	newly	20.	46.93	{5., 97.}	170.15	0.492197	Reff	1.27733	1.24596	{0.812056, 1.74375}	26.0262	ar	491.	640.55	{214., 907.}	55.5621
32	R0	{1.27601, 17.9804}	{1.60419, 13.0268}	{{1.0804, 5.32185}, {2.20181, 18.9813}}	{31.3176, 29.9555}	inf	14.	60.1249	{2.5376, 140.409}	359.169	0.624779	newly	3.	31.78	{3., 66.}	972.667	0.896811	Reff	1.24028	1.1353	{0.76354, 1.56866}	24.9248	ar	445.	564.09	{218., 815.}	50.5146
33	R0	{1.3368, 11.3235}	{1.80099, 13.3719}	{{1.11228, 5.72868}, {2.77502, 19.0269}}	{42.6579, 39.461}	inf	17.	73.4235	{3.79943, 176.883}	365.506	0.660415	newly	9.	36.41	{3., 78.}	330.778	0.666142	Reff	1.27531	1.26292	{0.764875, 1.96011}	31.1883	ar	417.	605.52	{219., 926.}	70.5516
34	R0	{1.21021, 11.3635}	{1.63675, 13.5868}	{{1.06836, 6.0993}, {2.24606, 19.0443}}	{39.328, 40.1904}	inf	11.	65.8987	{2.48733, 142.6}	525.406	0.744508	newly	11.	33.06	{1., 64.}	231.273	0.565501	Reff	1.12671	1.11809	{0.758662, 1.56866}	24.3341	ar	327.	575.26	{244., 861.}	89.0948
35	R0	{1.53382, 10.8866}	{1.78506, 13.2006}	{{1.11228, 6.88118}, {2.51743, 18.743}}	{31.7607, 38.7578}	inf	11.	74.4783	{4.99197, 159.976}	596.407	0.742057	newly	11.	41.73	{3., 91.}	305.545	0.59129	Reff	1.49547	1.34244	{0.904447, 1.91401}	25.1865	ar	476.	621.74	{172., 900.}	56.4286
36	R0	{1.20423, 6.83844}	{1.80738, 13.2993}	{{1.07874, 6.74711}, {2.55942, 19.0269}}	{53.5297, 97.7631}	inf	6.	62.2697	{3.4726, 176.077}	953.315	0.854704	newly	7.	35.8	{2., 82.}	436.	0.68579	Reff	1.18255	1.46766	{0.919739, 2.1477}	35.1488	ar	416.	627.77	{171., 889.}	75.5697
37	R0	{1.43629, 10.1548}	{1.65176, 13.6623}	{{1.11228, 6.31117}, {2.24606, 19.0443}}	{27.3511, 49.4474}	inf	10.	68.402	{2.57939, 147.091}	605.302	0.758895	newly	5.	34.47	{3., 67.}	605.4	0.775109	Reff	1.34006	1.12067	{0.762641, 1.54919}	26.7419	ar	367.	582.5	{244., 861.}	74.8447
38	R0	{1.37504, 8.93428}	{1.78985, 13.2872}	{{1.11164, 6.62607}, {2.52651, 19.1609}}	{38.8668, 58.4443}	inf	15.	62.5281	{3.69964, 160.648}	343.819	0.603211	newly	14.	35.93	{2., 82.}	189.929	0.520831	Reff	1.32829	1.45291	{0.919739, 2.03314}	28.0758	ar	483.	631.7	{207., 888.}	56.4969
39	R0	{1.51637, 9.36742}	{1.87997, 13.4321}	{{1.11927, 6.77341}, {2.6365, 18.1089}}	{34.6873, 54.928}	inf	28.	94.3758	{8.24928, 221.261}	265.365	0.560968	newly	20.	49.19	{6., 92.}	177.55	0.503676	Reff	1.37686	1.23293	{0.737199, 1.72673}	26.2273	ar	441.	677.41	{276., 907.}	69.5215
40	R0	{1.23658, 9.82869}	{1.94205, 13.5695}	{{1.12882, 6.55128}, {2.78218, 19.0269}}	{60.0042, 53.5216}	inf	23.	62.957	{4.78513, 168.293}	215.387	0.528423	newly	14.	38.08	{3., 89.}	211.857	0.523787	Reff	1.18712	1.62805	{1.04836, 2.23343}	43.0437	ar	414.	662.25	{137., 917.}	82.5193
41	R0	{1.24247, 5.4242}	{1.97229, 12.2726}	{{1.18939, 5.62019}, {2.80569, 18.9406}}	{61.0938, 129.346}	inf	9.	46.9126	{3.78379, 105.973}	445.337	0.683495	newly	11.	41.41	{3., 101.}	313.545	0.633236	Reff	1.22135	1.73387	{1.09441, 2.4542}	47.3427	ar	345.	653.25	{69., 928.}	113.487
42	R0	{1.28208, 4.40223}	{1.9314, 13.2845}	{{1.12882, 6.31117}, {2.79269, 19.0269}}	{54.7149, 203.598}	inf	3.	62.016	{4.78513, 153.674}	1973.57	1.0737	newly	6.	39.38	{3., 93.}	574.667	0.700043	Reff	1.26028	1.61356	{1.02997, 2.23343}	37.5552	ar	339.	658.47	{137., 917.}	113.667
43	R0	{1.56773, 6.50474}	{1.92944, 11.403}	{{1.25721, 5.3704}, {2.63959, 17.3398}}	{32.4736, 83.1747}	inf	6.	38.6362	{3.8895, 90.7609}	560.697	0.709351	newly	5.	44.97	{2., 102.}	819.	0.767706	Reff	1.55205	1.76102	{1.1496, 2.4171}	26.8036	ar	498.	661.49	{255., 902.}	53.7169
44	R0	{1.10489, 6.01941}	{1.8001, 14.9872}	{{1.24037, 9.28928}, {2.398, 20.}}	{62.9214, 151.03}	inf	18.	97.5654	{23.8308, 195.507}	442.48	0.607862	newly	22.	45.64	{13., 88.}	123.273	0.31187	Reff	1.03418	1.19139	{0.795975, 1.67075}	27.5243	ar	346.	787.38	{506., 1021.}	128.786
45	R0	{1.25888, 14.1926}	{1.66164, 14.9317}	{{1.23536, 8.26051}, {2.12482, 20.}}	{33.3725, 28.2566}	inf	42.	77.4766	{25.686, 153.877}	101.964	0.270168	newly	17.	36.89	{15., 63.}	123.	0.296644	Reff	1.09523	1.12263	{0.714598, 1.4955}	22.6924	ar	471.	754.77	{532., 987.}	61.0425
46	R0	{1.45292, 16.616}	{1.83072, 13.2759}	{{1.10693, 6.62607}, {2.64373, 18.8445}}	{37.1922, 24.9109}	inf	9.	60.6694	{3.8551, 155.006}	591.453	0.722857	newly	4.	34.85	{3., 80.}	785.25	0.847544	Reff	1.43258	1.50714	{0.961356, 2.17624}	27.4682	ar	494.	642.27	{236., 913.}	55.4474
47	R0	{1.22528, 16.6033}	{1.75459, 12.9367}	{{1.08887, 6.77341}, {2.46976, 18.743}}	{47.8634, 27.3671}	inf	12.	71.4317	{4.32503, 159.976}	516.932	0.701027	newly	3.	39.31	{3., 85.}	1220.33	0.969617	Reff	1.19955	1.308	{0.843719, 1.8965}	27.2381	ar	368.	604.62	{151., 890.}	86.5435
48	R0	{1.1669, 6.92715}	{1.67402, 14.6559}	{{1.20185, 7.04311}, {2.12528, 20.}}	{43.5541, 117.293}	inf	9.	79.457	{24.8661, 154.717}	782.855	0.848247	newly	10.	37.53	{15., 67.}	276.5	0.511071	Reff	1.05138	1.113	{0.76001, 1.53808}	23.1089	ar	416.	759.09	{492., 991.}	83.0937
49	R0	{1.32749, 16.0887}	{1.72466, 12.9678}	{{1.11461, 5.50601}, {2.67299, 18.9813}}	{37.0551, 27.9794}	inf	11.	66.6866	{3.85453, 162.199}	532.119	0.732259	newly	7.	35.15	{3., 80.}	423.571	0.701494	Reff	1.29165	1.24312	{0.771672, 1.94067}	29.294	ar	353.	588.25	{220., 907.}	86.3711
50	R0	{1.29636, 15.2609}	{1.75898, 14.7557}	{{1.23927, 8.01821}, {2.48836, 20.}}	{37.5744, 22.586}	inf	39.	91.7651	{29.0473, 184.398}	147.115	0.335564	newly	16.	43.52	{16., 83.}	174.75	0.373248	Reff	1.16283	1.13512	{0.657732, 1.52189}	22.6111	ar	470.	771.04	{480., 1018.}	66.5532
